# Investigation of Dynamic Behavior of Confined Ionic Liquid [BMIM]^+^[TCM]^−^ in Silica Material SBA-15 Using NMR

**DOI:** 10.3390/ijms24076739

**Published:** 2023-04-04

**Authors:** Lydia Gkoura, Nikolaos Panopoulos, Marina Karagianni, George Romanos, Aris Chatzichristos, George Papavassiliou, Jamal Hassan, Michael Fardis

**Affiliations:** 1 Institute of Nanoscience & Nanotechnology, NCSR Demokritos, Aghia Paraskevi, 15310 Athens, Greece; 2 Division of Science, New York University Abu Dhabi, Abu Dhabi 129188, United Arab Emirates; 3 Department of Physics, Khalifa University of Science and Technology, Abu Dhabi 127788, United Arab Emirates

**Keywords:** confined ionic liquids, NMR, relaxation and diffusion, mesoporous silica SBA-15

## Abstract

The molecular dynamics of 1-butyl-3-methyl imidazolium tricyanomethanide ionic liquid [BMIM]^+^[TCM]^−^ confined in SBA-15 mesoporous silica were examined using ^1^H NMR spin-lattice (T_1_) relaxation and diffusion measurements. An extensive temperature range (100 K–400 K) was considered in order to study both the liquid and glassy states. The hydrogen dynamics in the two states and the self-diffusion coefficients of the cation [BMIM]^+^ above the glass transition temperature were extracted from the experimental data. The results were then compared to the corresponding bulk substance. The effects of confinement on the dynamic properties of the ionic liquid clearly manifest themselves in both temperature regimes. In the high-temperature liquid state, the mobility of the confined cations reduces significantly compared to the bulk; interestingly, confinement drives the ionic liquid to the glassy state at a higher temperature *T*_g_ than the bulk ionic liquid, whereas an unusual T_1_ temperature dependence is observed in the high-temperature regime, assigned to the interaction of the ionic liquid with the silica-OH species.

## 1. Introduction

Water and several other liquids have been studied under geometrical confinement for decades, and their behavior can be considered reasonably well understood [1]. Confined liquids are subject to two concurring effects: the presence of the surface and the geometrical aspect of the restricted space itself [2]. While the latter affects thermodynamic properties, such as melting point and glass transition temperatures, the former possesses the potential, depending on the nature of the solid surface, to enhance or reduce molecular order and restrict or even promote mobility on a molecular scale [3].

Room temperature ionic liquids (ILs) are a class of organic salts that consists of a variety of cations and anions. ILs are liquids at room temperature with fascinating properties depending on the type of cation or anion used to make them. They have unique characteristics that make them attractive in many applications ranging from energy storage and production devices, such as batteries [3,4] and super capacitors [5], to gas absorption systems [6]. ILs are used as bulk or incorporated within materials with a high surface to volume ratio, such as porous materials. The ordering and mobility of ILs within (nano)pores show significant enhancement in catalytic activity [7], charge transport, and electrical conductivity [8,9]. Therefore, immobilization of ILs in solid porous materials renders them more suitable for a broad range of applications.

It has been proposed that the confinement changes the cation–anion distances, which in turn affect their properties [10]. Therefore, understanding the transport properties as well as the ionic and solvent interactions of ILs enables the prediction of their physicochemical properties. The impact of structure, molecular organization, and cation–anion interactions on the physical properties of ILs is still a topic of major research interest [1,11]. Furthermore, many ILs belong to the class of glass-forming materials, exhibiting a transition from the liquid to a rigid glassy phase at a transition temperature *Tg*, whose microscopic origin of the temperature dependence of structural and dynamic properties is still not well-understood [12,13,14].

Under this context, a great number of experimental and theoretical efforts have been employed in order to examine guest–host molecular systems [15,16,17], including structural relaxation times and transport coefficients of confined glass-forming materials [18]. From the experimental point of view, the dynamical properties of many ILs have been investigated with several techniques, such as dielectric spectroscopy [19,20], neutron scattering [21,22,23,24], light scattering [19], and NMR spectroscopy [17,25,26,27,28,29,30,31,32]. Theoretical studies using Molecular Dynamics (MD) simulations also reveal complex phase transitions of nano-confined ILs [32,33,34]. We refer readers to Ref. [35], which includes detailed information on various experimental studies dealing with the changes in the physicochemical properties (such as thermal phase transition, stability, dynamical behavior, and optical properties) of ionic liquids confined inside porous materials.

In a recent publication [32], we combined MD simulations with ^1^H NMR and showed that [BMIM]^+^[TCM]^−^ entrapped into the narrow MCM-41 silica nanopores exhibited a complex dynamic ordering and stratified diffusion close to single file diffusion in the liquid state high-temperature regime. This effect was not observed in the wider SBA-15 nanoporous silica, where almost normal diffusion was detected.

It should be stressed that many studies have investigated the dynamics of ILs in confined geometries of silica materials [36,37,38], carbon nanotubes [39], and porous polymer monoliths [40]. However, most of them (with few exceptions using mainly dielectric relaxation spectroscopy [41,42]) examined the IL mobility within the high-temperature phase above the glass transition temperature. This is due to the fact that all translation degrees of freedom of the ILs freeze out at low temperatures within the glassy state, making the macroscopic observables (self-diffusion coefficients and viscosity) difficult to measure.

In this work, we report ^1^H NMR relaxation and 2D NMR diffusion measurements of IL (1-butyl-3-methyl imidazolium tricyanomethanide ionic liquid [BMIM]^+^[TCM]^−^) in bulk as well as confined in SBA-15 (average pore size of 6–11 nm) mesoporous materials. The measurements cover a wide range of temperatures (100 K–400 K) to obtain information on the molecular dynamics of this IL in both the liquid and the glassy states (above and below the glass transition temperatures for bulk and confined IL [43]).

## 2. Results and Discussion

### 2.1. T_1_ Relaxation Time Measurements

**Figure 1:**
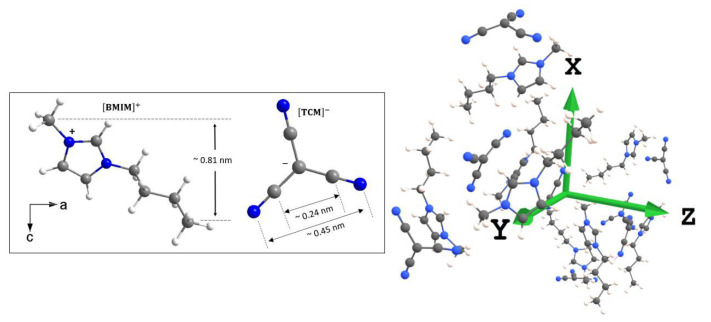
**Left**: A single molecule of the ionic liquid [BMIM]^+^[TCM]^−^ used in this study. The [BMIM]^+^ cation is the 1-butyl-3-methylimidazolium (C_8_H_15_N_2_) molecule, and the [TCM]^−^ is the tricyanomethanide (C_4_N_3_) molecule. **Right:** The molecular arrangement of a cluster of IL molecules optimized using the ORCA software package [44,45]. Blue, grey, and white circles are nitrogen, carbon, and hydrogen atoms, respectively.

Figure 1 shows the structure of a cluster of [BMIM]^+^[TCM]^−^ IL molecules used in this study (with their geometry optimized using ORCA software). As can be observed, the [BMIM]^+^ cation contains fifteen ^1^H nuclei of which six belong to the two methyl groups. One is attached to the end of the alkyl chain, and the other is attached to the imidazolium of the cation. These groups contribute to approximately half (or specifically 6/15) of the ^1^H NMR signal, both for the bulk IL and the IL confined inside SBA-15.

NMR relaxation times are determined by the fluctuations of the local magnetic fields inside the material, which are modulated by the molecular motions activated by the thermal energy of the molecules. Therefore, NMR spin-lattice relaxation time *T*_1_ or spin-spin relaxation time *T*_2_ are important in order to acquire direct information on the molecular dynamics. For example, observation of an NMR *T*_1_ minimum on the *T*_1_ versus temperature curve gives a direct measurement of the characteristic correlation time $\tau_{c}$ of the molecular motion through the well-known NMR relationship $\omega_{L}\tau_{c}\sim 1$, where $\omega_{L}$ is the Larmor angular frequency for the active NMR nuclei [46], in this case, ^1^H.

**Figure 2:**
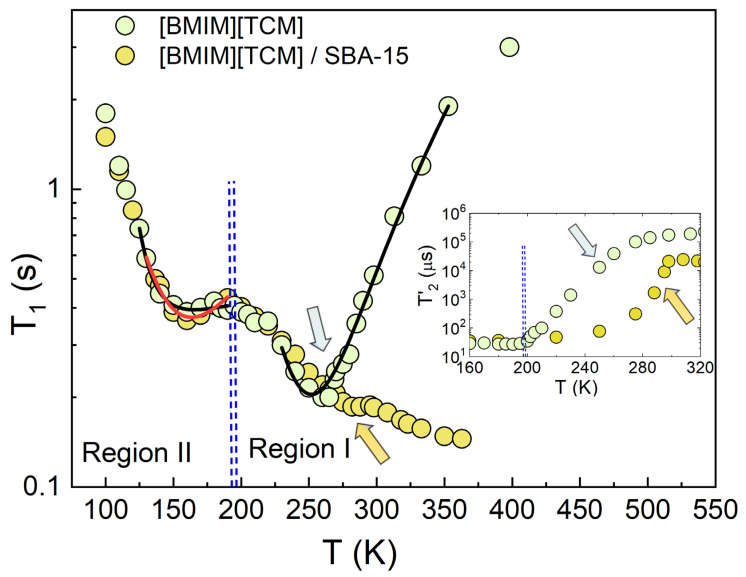
Spin-lattice *T*_1_ relaxation time as a function of temperature for the bulk IL and confined IL in SBA-15. The two vertical dashed lines are the glass transition temperatures for the bulk ($T_{g}=186.4\;K$) and confined ILs ($T_{g}=190.5\;K$), obtained using the DSC technique. The arrows indicate the temperatures at which T_1_ minima occur above *T*_g_ (Region I). The solid curves are the theoretical fits to the experimental data using the BPP model (refer to the text). The inset of the figure shows the spin-spin relaxation time $T_{2}$ versus temperature.

**Figure A1:**
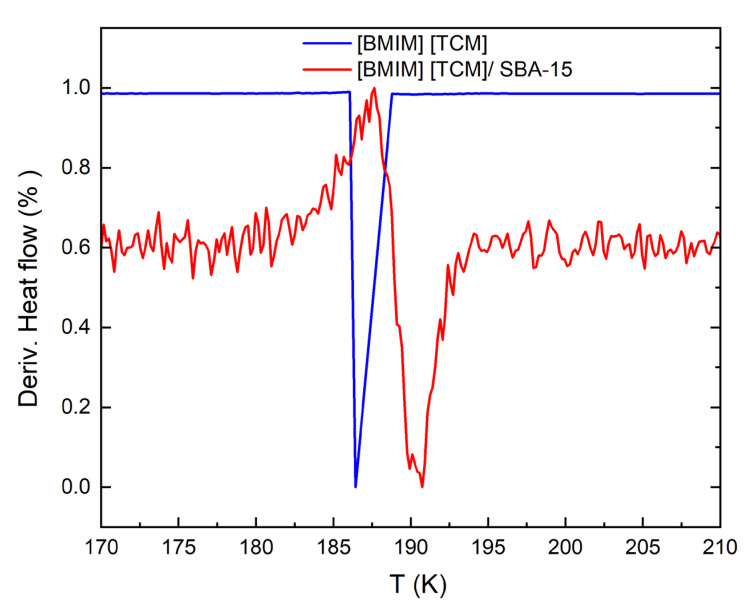
Results of the derivatives of the heat flow in the relevant Differential Scanning Calorimetry (DSC) experiments. As observed, there is a difference of approximately 4.1 K between the two glass transition temperatures (*Tg* for the bulk IL is 186.4 K and *Tg* for the confined IL inside SBA-15 is 190.5 K).

**Figure A2:**
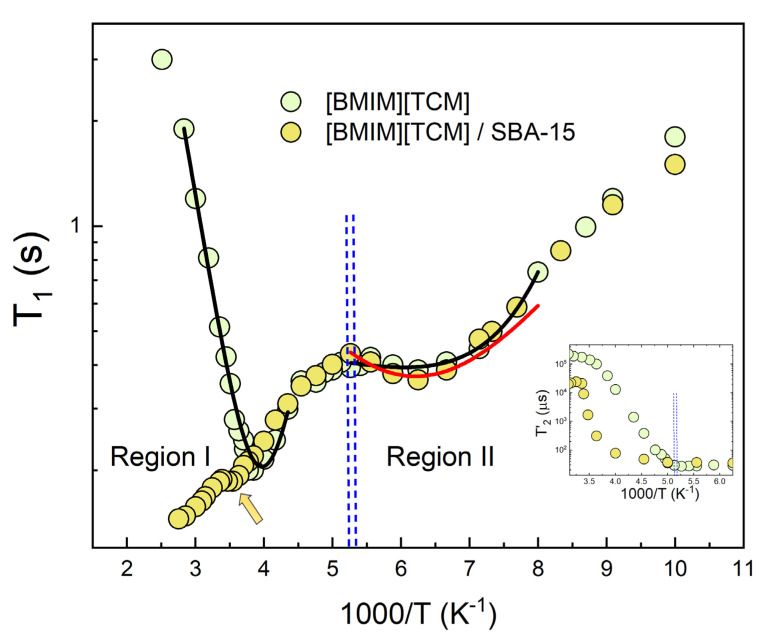
Replicate of the data shown in Figure 2 of the main text. Here, spin-relaxation times are shown versus reciprocal of temperature for both the bulk and confined ionic liquids. The lines are fit using the BPP model (refer to the main text for details). The two vertical dashed lines are the glass transition temperatures for the bulk (*Tg* = 186.4 K) and confined ILs (*Tg* = 190.5 K) obtained using the DSC technique (shown in Figure A1). The inset of the figure shows the spin-spin relaxation times $T_{2}$ vs. T.

Figure 2 shows the ^1^H spin-lattice *T*_1_ relaxation time of the bulk and confined [BMIM]^+^[TCM]^−^ IL as a function of temperature. Region I and region II represent the temperature ranges above and below the glass transition temperature for bulk IL [43]. The glass transition temperatures for the bulk and confined ionic liquids were measured using differential scanning experiments (Appendix A, Figure A1), where *T_g_* for bulk IL is 186.4 K, and *T_g_* for confined IL inside SBA-15 is 190.5 K. The spin-relaxation times versus reciprocal temperature are also shown in the Appendix A, Figure A2.

At the low-temperature region, *T*_1_ minima are observed for both liquids at ~160 K. In addition, the data show a well-defined *T*_1_ minimum at high T for the bulk IL and a less pronounced *T*_1_ minimum for the confined IL (shown with arrows in Figure 2). The inset of the figure shows the spin-spin $T_{2}$ relaxation time versus temperature. The decrease in the $T_{2}s$ for both the bulk and confined ILs reflects the freezing of the translational motion; remarkably, in the case of the confined IL, freezing starts at a sufficiently higher temperature than the bulk IL.

It is important to note that the OH protons on the surface of the silica materials have long T_1_ (4.0 s–5.0 s) and very short T_2_ (40 μs–50 μs). Therefore, in our relaxation measurements, precautions were taken to filter out the proton signal from the silica surface OH groups by using appropriate pulse repetition times and Hahn SE interpulse times, particularly at high temperatures where the relaxation times for IL and surface OH protons are significantly different from each other. Besides, the OH proton NMR signal is very weak, as confirmed in Figures 2 and 4 of our recently published work [32], and therefore, it is not expected to influence the ^1^H NMR measurements at low temperatures, where T_1_ and T_2_ values of OH are of the same order of magnitude as those of the ILs. This fact is supported by the similarity of the T_1_ vs. T curves at low temperatures for both the bulk IL (where there are no surface OH protons) and the confined IL/SBA-15.

On the basis of the relationship $\omega_{L}\tau_{c}\sim 1$, the correlation times $\tau_{c}$ of the bulk and confined ILs are in the sub-microsecond time scale. Nevertheless, a careful analysis of the experimental data is required for a more accurate characterization of the molecular dynamics. One model that is routinely used in fitting *T*_1_ versus temperature data is the model proposed by Bloembergen, Purcell, and Pound [47] (BPP). In this model, the spin-lattice relaxation rate $1/T_{1}$ depends on the spectral densities $J\left(\omega_{L}\right)$ and $J\left(2\omega_{L}\right)$ of the local magnetic fluctuations at the Larmor frequency, $\omega_{L}$, through the following formula:

$$\frac{1}{T_{1}}=2K\;\frac{\gamma^{4}}{r^{6}}\;\left[J\left(\omega_{L}\right)+4\;J\left(2\omega_{L}\right)\right]$$

$$\frac{1}{T_{1}}=\frac{3}{10}\;\;\frac{\hbar^{2}\;\gamma^{4}}{r^{6}}\;[\frac{\tau_{c}}{1+\omega_{L}^{2}\;\tau_{c}^{2}}+\frac{4\tau_{c}}{1+4\omega_{L}^{2}\;\tau_{c}^{2}}]$$

Here, *τ*_c_ denotes the correlation time characteristic of the random motion, ℏ is the reduced Plank’s constant, γ is the gyromagnetic ratio, and r is the distance between two interacting dipoles.

The ^1^H relaxation mechanism in bulk IL at high temperatures, where diffusion is rapid, is controlled by the diffusional modulation of the inter-nuclear dipolar fields as proposed in the BPP model. Consequently, the bulk IL *T*_1_ data in region I were fitted to the BPP model (solid black line shown in Figure 2) assuming that the diffusion process is thermally activated, i.e., $\tau =\tau_{0}exp\left(E_{a}/k_{B}T\right)$ where *E_a_* is the activation energy for the tumbling motion of molecules under investigation, and $\tau_{o}$ is the characteristic time of the molecular motion. We refer to the molecular motion as tumbling because, at high temperatures, it is expected that both translational and rotational processes are coupled together. The solid curve in region I of Figure 2 was obtained by using the most probable value for *τ_c_*, which is in good agreement with previously published values in similar [BMIM]^+^ based ionic liquids [12,21,30]. The average correlation time *τ*_c_ at the *T*_1_ minimum at 250 K was calculated to be 1.0 ns.

It is worth noting that, above 260 K, the temperature dependence of *T*_1_ for the bulk IL is different from that in the confined IL. In bulk IL, the *T*_1_ values increase with increasing temperature, while the opposite change is observed for the *T*_1_ data of the confined ILs. The reason is that, at high temperatures, the molecules of bulk IL are moving rapidly with short correlation times where $\tau_{c}\ll 1/\omega_{L}$. In this case, there are some limited spectral densities at $\omega_{L}$ and $2\omega_{L}\;$ with their low amplitudes stemming from the fact that the energy of the molecular motion is spread over a very broad frequency range. As a result, relaxation times increase with temperature (as observed from the data of bulk IL), in line with what is expected by BPP theory. Additionally, considering spin-spin interaction (intramolecular and intermolecular interactions) among all the spin pairs, the relaxation is expected to vary with self-diffusion coefficients D as $1/T_{1}{}_{Bulk}$ ∝ 1/D [48] with the explicit equation given as:

$${\left(\frac{1}{T_{1}}\right)}_{translation/Bulk\;}=\frac{\pi }{5}\frac{N\gamma^{4}\;\hbar^{2}}{a\;D}\;\;\propto \frac{1}{D}$$

where a represents the molecular size, ℏ is the reduced Plank’s constant, and γ is the gyromagnetic ratio of the spins. As we will observe later (Figure 5), at high temperatures and bulk IL, the D is proportional to T, similar to T_1_ as observed in Figure 2.

On the other hand, the pore walls and the surface interactions affect the relaxation mechanism for confined molecules inside the silica material. In this case, the relaxation rate is considered to be proportional to the self-diffusion coefficient of the molecules, $1/T_{1}{}_{wall}$ ∝ D [49]. This is because the increase in parameter D, as a response to increasing temperature, enables the molecules to diffuse faster and reach the surface of the pores more often. These molecules then quickly relax due to the presence of unpaired electrons from the oxygen groups on the SBA-15 silica walls [50] (the density of Si-O-OH bonded OH groups on the surface of SBA-15 was reported to be approximately 3.7 OH/nm^2^ [51]). This scenario corroborates with the fact that a layer of adsorbed IL molecules on the surface of the SBA-15 at room temperature decreases gradually with increasing temperature [32], as also shown later in Figure 4.

The *T*_1_ minimum at the low-temperature regime (region II, i.e., below *T*_g_, refer to Figure 2) indicates a type of molecular motion with frequency in the MHz region within the glassy state. We attribute this to the motion of the methyl groups of the IL. It is well known that methyl rotation is the fastest dynamic in organic systems. Therefore, it is expected that this rotation mechanism manifests itself within the low-temperature glassy state. The *T*_1_ minimum in the low-temperature region II can thus be ascribed to the three-fold rotation of the two methyl groups with respect to their C-C bond, where the characteristic correlation time of this motion is comparable to the NMR Larmor period.

Furthermore, ^1^H NMR spin-spin relaxation time measurements in the low-temperature regime of region II (inset of Figure 2 and ref. [32]) appear to be temperature independent. This is because, at low temperatures, all the translational motions of the IL cations are frozen out. The only motion surviving is the reorientation of the methyl groups in agreement with previously reported results using NMR techniques in polymers and organic compounds (refer to, for example, Refs. [52,53,54,55]).

The experimental *T*_1_ data for bulk IL at low temperatures between 130 K to 190 K were fitted to the BPP model (Figure 2), giving an activation energy $E_{a}$ for the methyl hindered rotation of approximately 5.0 kJ/mol. The low value of the $E_{a}$ of 5.0 kJ/mol in region II compared to 22 kJ/mol in region I for bulk IL signifies the difference in the origin of the molecular motion of the IL in the low- and the high-temperature regimes. It is frequently observed that the activation energy for methyl-group rotation, determined from proton *T*_1_ measurements, changes to a lower value as the temperature is reduced [56]. In the high-temperature phase, the motion of the whole molecule requires higher activation energy than the one needed for the rotational motion of methyl groups at the low-temperature regime (refer to, for example, Refs. [57,58]). In addition, in the glassy state, the methyl groups populate different local environments due to the structural disorder inherent to the glassy state. This manifests itself in different rates of rotation [59]. The activation energies $E_{a}\;$ for the methyl rotations of the confined ILs at low temperatures (region II) were also calculated using the Arrhenius activation form and were found to be equal to 5.8 kJ/mol, close to the value found for the bulk IL (5.0 kJ/mol). This indicates that the methyl rotation is not significantly perturbed by the confinement effect. Finally, both factors ($\tau_{o},\;T$) contribute to the higher activation energy of the molecular motion in region I compared to that in region II. This is expected because, at the high-temperature region I, the whole molecules are in motion, and therefore, more energy is required to activate motions in comparison to the low-temperature region II where only methyl group motion persists (which, of course, requires a smaller activation energy).

### 2.2. Self-Diffusion in the High-Temperature Regime

It is well known that experiments to measure the self-diffusion coefficient *D* using NMR spectroscopy in the presence of a static or pulsed magnetic field gradient can directly determine the mobility at the atomic and molecular levels [60]. The diffusion coefficients obtained in this way do not depend on any assumptions about microscopic details of the atomic motion, such as jump length or hard-core dimensionality (e.g., Einstein−Smolouchouski’s law [61]); therefore, the measurements are purely hydrodynamic in nature.

In the conventional 1D NMR diffusion experiments with a uniform diffusion process, *D* is obtained by appropriately fitting the ^1^H NMR spin-echo decay data [62]. However, in the case of non-uniform diffusion processes (e.g., liquids confined in pore structures), diffusion is expressed with a distribution function $f\left(D\right)$. This function is obtained by implementing an appropriate 1D inversion algorithm on the spin-echo decay data [63].

**Figure 3:**
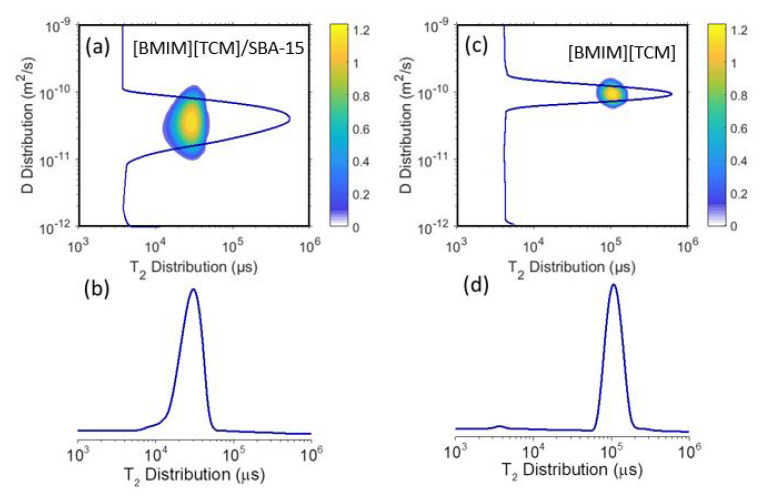
(**a**,**c**) 2D ^1^H NMR *D-T*_2eff_ contour plots of the bulk ionic liquid and confined [BMIM]^+^[TCM]^−^ ionic liquid in SBA-15 at 292 K. (**b**,**d**) Distribution of the *T*_2eff_ values obtained from the 2D contour plots.

Alternatively, 2D diffusion-relaxation D-T_2eff_ correlation spectroscopy is advantageous compared to 1D NMR diffusion experiments, as it allows the measurement of spin-spin relaxation times (T_2eff_) with the relevant D values [64]. Here, the D-T_2eff_ technique has been employed to examine the relevant differences between the T_2eff_ and D values of the bulk and confined ILs. Figure 3 shows the representative 2D contour results for the bulk and confined ILs at 292 K.

The result for the bulk IL shows a narrow region on the 2D plot represented by a single *D* value of~1 × 10^−10^ m^2^/s and a single *T*_2_ value of~8 × 10^4^ μs at 292 K. Narrow peaks in *D* and *T*_2_ windows are expected for bulk liquids. On the other hand, the data of the confined IL inside SBA-15 show a remarkably broader distribution of the D values and a relatively broader *T*_2_ distribution, as observed in the 2D plots in Figure 3. The broadening of the *D* peak for the confined IL inside SBA-15 compared to the bulk IL is mainly because the molecules close to the pore walls are getting a slower diffusion, whilst those at the pore center acquire almost bulk-like diffusion.

**Figure 4:**
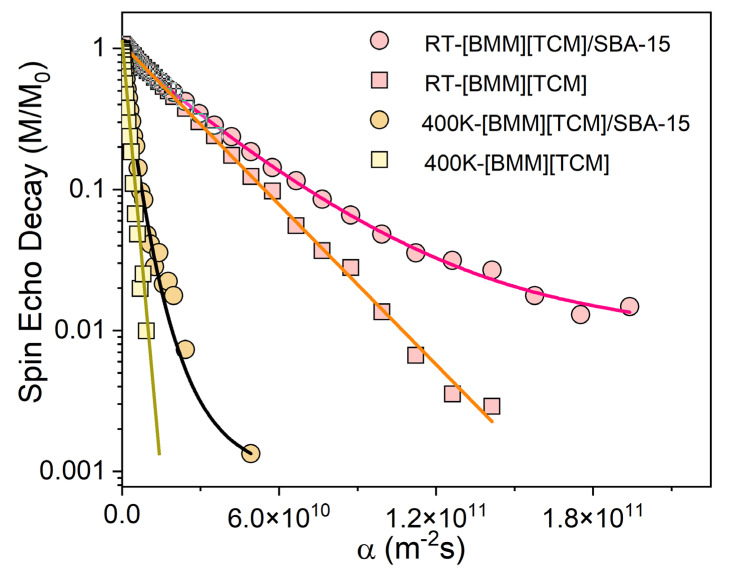
^1^H NMR SED plots of the bulk ionic liquid and confined [BMIM]^+^[TCM]^−^ ionic liquid in SBA-15 at room temperature and 400 K at the Larmor frequency of 100 MHz and in a constant magnetic field gradient of G = 34.7 Tesla/m. In the case of a homogeneous diffusion process with a single self-diffusion coefficient D, the spin echoes decay according to the law M/M0 = exp(−D α), where α = (2/3)γ^2^G^2^τ^3^.

This is best observed in Figure 4, which presents the 1H NMR Hahn spin-echo decays (SEDs) of the two samples at the Larmor frequency of 100 MHz and in a constant magnetic field gradient of G = 34.7 Tesla/m at RT and 400 K.

In the case of a homogeneous diffusion process with a single self-diffusion coefficient *D*, SEDs decay according to the law M/M_0_ = exp(−D α), where α = (2/3)γ^2^G^2^τ^3^. Here γ is the gyromagnetic ratio of ^1^H. Indeed, in the case of the bulk IL, a single D value is obtained with the values D = 6 × 10^−11^ m^2^/s at RT and 5 × 10^−10^ at 400 K. However, in the case of the IL inside the SBA-15 nanopores, the slope deviates to lower values as α gets larger, showcasing the presence of a distribution of D-values, which decrease as the IL gets closer to the silica walls, in accordance with Figure 3a. Notably, by comparing the slopes of the bulk and confined ILs, it is observed that, at room temperature, almost 50% of the confined IL behaves as bulk IL (they have the same slope, i.e., D values), while at 400 K, the bulk-like part increases to 80%, indicating that the adsorbed slow-diffusing IL layer becomes thinner with increasing temperature.

**Figure 5:**
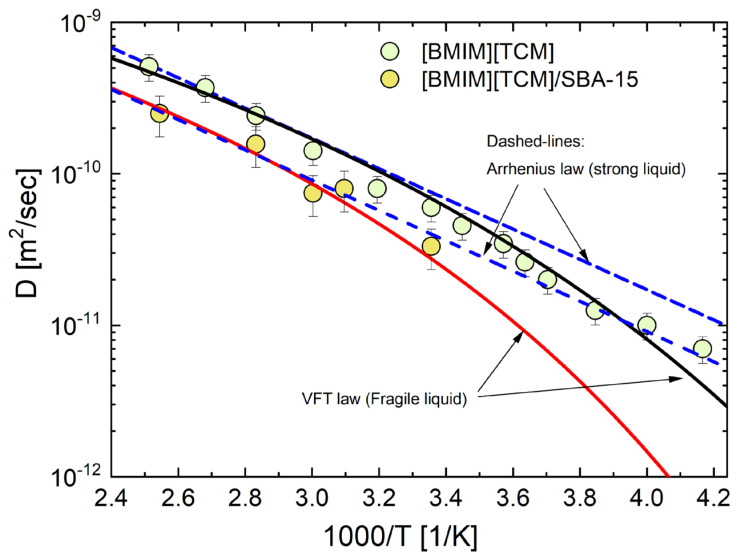
Self-diffusion coefficients *D* as a function of the inverse temperature obtained from ^1^H NMR measurements for the bulk ionic liquid and confined [BMIM]^+^[TCM]^−^ ionic liquid in SBA-15. The black and red lines are the fit curves obtained using the VFT law. The dashed lines are the relevant Arrhenius curves.

In order to study the evolution of the diffusion dynamics in the high-temperature regime, D-T_2eff_ experiments were performed in the temperature range 240 K–400 K, and the average log-mean D values at each temperature were extracted, as shown in Figure 5, exhibiting the temperature dependence of the relevant D values.

In the literature, different models have been considered for the data analysis using the concept of fragility introduced by Angel [65], who classified a liquid as a strong liquid or a fragile liquid. This classification is based on whether the liquid’s temperature dependence of a dynamic observable, such as self-diffusion coefficients or structural relaxation times, follows an Arrhenius or a non-Arrhenius behavior. A pure Arrhenius behavior classifies a strong liquid, whereas a non-Arrhenius one signifies a fragile behavior. For example, liquid water is a fragile liquid because the temperature dependence of its thermodynamic properties does not obey the Arrhenius law.

For the IL considered in this study, it appears that *D* varies in a non-Arrhenius way with temperature, at least for the bulk IL, indicating a fragile behavior. This is further observed by comparing the statistical parameters obtained from the fittings, shown in Table A1 in Appendix A. Therefore, we have used a Vogel–Fulcher–Tammann (VFT) law $D=D_{0}exp\left[\frac{-B}{\left(T-T_{0}\right)}\right]$ to fit the diffusion data [66]. The solid black and red curves are the best fit curves using the VFT law, shown in Figure 5. The dashed lines depict the relevant Arrhenius fits, while it is evident that the bulk IL data do not conform to the curve. In the VFT law, the parameter B represents the strength of the activated process, which governs the flow behavior of the material. A lower value of B indicates that the glassy liquid is more fragile in nature. The VFT law diverges at the finite temperature *T*_0_, where the configurational entropy vanishes [67]. Similar values of *B* and *T*_0_ were estimated for the bulk IL (810 K, 137 K) and the confined IL/SBA-15 (800 K, 157 K) systems. The adjustable parameters are in agreement with reported values for the [BMIM] cation [25].

In the case of the confined IL, both fitted curves (Arrhenius and VFT) are substantially in line with the data, yet it appears that the VFT law provides a better representation (refer to Table A1, Appendix A). The reason for this is that the temperature range examined herein is restricted, primarily due to the freezing of the confined IL at relatively high temperatures. Therefore, in this case, no conclusive inference can be drawn on the fragility of the confined liquid. Further research is required in this regard.

Finally, it is observed that, in the high-temperature liquid state, the mobility of the confined cations reduces significantly compared to that of the bulk IL as revealed by the lower values of the self-diffusion coefficients. This is in accord with results reported in the literature [31,68].

## 3. Materials and Methods

### 3.1. Chemicals and Porous Materials

The [BMIM]^+^[TCM]^−^ (mass fraction purity >98% with 720 ppm H_2_O content) was purchased from IoLiTech GmbH. Mesoporous SBA-15 with cylindrically shaped pores [69] (nominal pore size 6–11 nm) was obtained from ACS Material. Prior to sample preparations, the silica powder was dried overnight at 100 °C under a weak pump system to remove any possible water vapor within the material. The procedure for pore filling with the IL was previously reported in ref. [32].

### 3.2. NMR Spectroscopy

NMR relaxation experiments were performed at 100.123 MHz using a broadband pulse NMR spectrometer. For the spin-lattice relaxation time *T*_1_ measurements, the typical NMR inversion recovery pulse sequence (180°-τ-90°-signal-) was used. The 90° pulse-width was set to 3.0 μs; the inter-pulse duration was 10–15 μs with a repetition time of 6.0 s. For the spin-spin relaxation time *T*_2_ measurements, the Hahn spin-echo (SE) pulse sequence was used, 90°-τ-180°-τ-SE (90° pulse width was set to 3.0 μs). 2D NMR diffusion-relaxation *D*-*T*_2eff_ experiments were conducted in the fringe field of a 4.7 T superconducting magnet providing a 34.7 T/m linear magnetic field gradient (the details of the pulse sequence can be found in Refs. [15,32,64]).

An Oxford 1200CF continuous flow cryostat was employed for the temperature measurements in the range between 100 K to 400 K. The accuracy of the temperature was ±1.0  K. The samples were initially cooled down to 100 K, and the NMR measurements were obtained on a heating cycle. This was followed because it is well known that certain ILs exhibit a notable hysteresis behavior, for example, in their ionic conductivity [70] and in their NMR relaxation measurements [71,72].

### 3.3. Software

To optimize the structure of the molecules of the IL used in this study, the free and open-source software ORCA (Open-Source Computational Chemistry Software) was used. This software utilizes a combination of ab initio, density functional theory, and semi-empirical methods to perform calculations and optimization of molecular systems. It can handle a variety of molecular systems, ranging from small to large molecules. Full documentation about this software can be found in the original references [44,45].

## 4. Conclusions

Measurements were carried out over a wide range of temperatures (100 K–400 K), covering the bulk IL and confined IL dynamics below and above their glass transition points, 186.4 K and 190.5 K, respectively. In the high-temperature regime (above *T*_g_), the dynamical behavior of the IL molecules depends on the molecular tumbling, reorientations of proton groups, and translational motions associated with self-diffusion. It was observed that, in the bulk ionic liquid, *D* follows a VFT temperature dependence indicative of a fragile liquid behavior towards the glass transition temperature. In this case, the underlying motion is predominately translational diffusion motion. On the other hand, the *T*_1_ relaxation data also revealed re-orientational motions that predominately originated from the rotation of the two CH_3_ groups of the IL molecules. These methyl rotations manifest themselves clearly by the appearance of the *T*_1_ minimum in the low-temperature regime below *T*_g_. In this regime, all translational degrees of freedom have frozen, while the methyl group rotation persists even below *T*_g_.

The effect of confinement on the dynamic properties of the ionic liquid clearly manifests itself in both temperature regimes. In the high-temperature liquid state, the mobility of the confined cations reduces significantly compared to that of the bulk IL as revealed by the lower values of the self-diffusion coefficients. The *T*_1_ relaxation data are highly influenced by the presence of pore walls of the mesoporous materials, especially at high temperatures.

We believe that further research is warranted to extend the results of this study and suggest the following.

First, obtain additional experimental self-diffusion data points over a wider temperature range than what is presented in the current work. This will provide a definitive conclusion as to whether the temperature evolution of the diffusion rate of confined IL follows an ideal- or fragile-liquid behavior. Second, conduct further theoretical work, such as MD simulations, to investigate the dynamics of IL molecules on the silica surface of pure SBA-15, which only contains OH groups on its surface, and SBA-15 with impurities represented by metal ions versus temperature. This work could then provide deeper insight into the origins of the anomalous dynamics of confined IL compared to bulk IL, especially at high temperatures above the glass transition.

## Data Availability

Not applicable.

## Appendix A

**Table A1:** Statistical parameters obtained from fitting the self-diffusion data vs. 1000/T for the bulk and confined ILs using the Arrhenius and VFT models.

	Bulk IL		Confined IL	
	Arrhenius	VFT	Arrhenius	VFT
Reduced X^2^ Adjustable R^2^ F-value RSS *	5.9 × 10^−22^ 0.995 271 5.9 × 10^−21^	1.28 × 10^−22^ 0.976 2343 1.4 × 10^−21^	8.2 × 10^−25^ 0.971 18.6 8.2 × 10^−22^	5.2 × 10^−22^ 0.93 63.9 1.03 × 10^−21^

* RSS: Residual sum of squares.

## References

[1] Buntkowsky G., Vogel M. (2020). Small Molecules, Non-Covalent Interactions, and Confinement. Molecules.

[2] He F., Wang L.-M., Richert R. (2007). Confined Viscous Liquids: Interfacial versus Finite Size Effects. Eur. Phys. J. Spec. Top..

[3] Gupta A.K., Verma Y.L., Singh R.K., Chandra S. (2014). Studies on an Ionic Liquid Confined in Silica Nanopores: Change in *T* _g_ and Evidence of Organic–Inorganic Linkage at the Pore Wall Surface. J. Phys. Chem. C.

[4] Broussely M., Biensan P., Bonhomme F., Blanchard P., Herreyre S., Nechev K., Staniewicz R.J. (2005). Main Aging Mechanisms in Li Ion Batteries. J. Power Sources.

[5] Balducci A., Yin Z., Zhang X., Tu Z., Hu X., Wu Y. (2004). Ionic Liquids for Hybrid Supercapacitors. Electrochem. Commun..

[6] Shi W., Maginn E.J. (2008). Molecular Simulation and Regular Solution Theory Modeling of Pure and Mixed Gas Absorption in the Ionic Liquid 1- *n* -Hexyl-3-Methylimidazolium Bis(Trifluoromethylsulfonyl)Amide ([Hmim][Tf _2_ N]). J. Phys. Chem. B.

[7] Craythorne S.J., Anderson K., Lorenzini F., McCausland C., Smith E.F., Licence P., Marr A.C., Marr P.C. (2009). The Co-Entrapment of a Homogeneous Catalyst and an Ionic Liquid by a Sol-Gel Method: Recyclable Ionogel Hydrogenation Catalysts. Chem. Eur. J..

[8] Iacob C., Sangoro J.R., Kipnusu W.K., Valiullin R., Kärger J., Kremer F. (2012). Enhanced Charge Transport in Nano-Confined Ionic Liquids. Soft Matter.

[9] Sangoro J.R., Kremer F. (2012). Charge Transport and Glassy Dynamics in Ionic Liquids. Acc. Chem. Res..

[10] Li C., Guo X., He Y., Jiang Z., Wang Y., Chen S., Fu H., Zou Y., Dai S., Wu G. (2013). Compression of Ionic Liquid When Confined in Porous Silica Nanoparticles. RSC Adv..

[11] Borghi F., Podestà A. (2020). Ionic Liquids under Nanoscale Confinement. Adv. Phys. X.

[12] Rivera A., Brodin A., Pugachev A., Rössler E.A. (2007). Orientational and Translational Dynamics in Room Temperature Ionic Liquids. J. Chem. Phys..

[13] Richert R., Blumen A. (1994). Disorder Effects on Relaxational Processes.

[14] Huber P. (2015). Soft Matter in Hard Confinement: Phase Transition Thermodynamics, Structure, Texture, Diffusion and Flow in Nanoporous Media. J. Phys. Condens. Matter.

[15] Gkoura L., Diamantopoulos G., Fardis M., Homouz D., Alhassan S., Beazi-Katsioti M., Karagianni M., Anastasiou A., Romanos G., Hassan J. (2020). The Peculiar Size and Temperature Dependence of Water Diffusion in Carbon Nanotubes Studied with 2D NMR Diffusion–Relaxation D–T2eff Spectroscopy. Biomicrofluidics.

[16] Chatzichristos A., Hassan J. (2022). Current Understanding of Water Properties inside Carbon Nanotubes. Nanomaterials.

[17] Fardis M., Karagianni M., Gkoura L., Papavassiliou G. (2022). Self-Diffusion in Confined Water: A Comparison between the Dynamics of Supercooled Water in Hydrophobic Carbon Nanotubes and Hydrophilic Porous Silica. Int. J. Mol. Sci..

[18] Alcoutlabi M., McKenna G.B. (2005). Effects of Confinement on Material Behaviour at the Nanometre Size Scale. J. Phys. Condens. Matter.

[19] Griffin P.J., Agapov A.L., Sokolov A.P. (2012). Translation-Rotation Decoupling and Nonexponentiality in Room Temperature Ionic Liquids. Phys. Rev. E.

[20] Sippel P., Krohns S., Reuter D., Lunkenheimer P., Loidl A. (2018). Importance of Reorientational Dynamics for the Charge Transport in Ionic Liquids. Phys. Rev. E.

[21] Triolo A., Russina O., Hardacre C., Nieuwenhuyzen M., Gonzalez M.A., Grimm H. (2005). Relaxation Processes in Room Temperature Ionic Liquids: The Case of 1-Butyl-3-Methyl Imidazolium Hexafluorophosphate. J. Phys. Chem. B.

[22] Aoun B., González M.A., Ollivier J., Russina M., Izaola Z., Price D.L., Saboungi M.-L. (2010). Translational and Reorientational Dynamics of an Imidazolium-Based Ionic Liquid. J. Phys. Chem. Lett..

[23] Kofu M., Tyagi M., Inamura Y., Miyazaki K., Yamamuro O. (2015). Quasielastic Neutron Scattering Studies on Glass-Forming Ionic Liquids with Imidazolium Cations. J. Chem. Phys..

[24] Mitra S., Cerclier C., Berrod Q., Ferdeghini F., de Oliveira-Silva R., Judeinstein P., le Bideau J., Zanotti J.-M. (2017). Ionic Liquids Confined in Silica Ionogels: Structural, Thermal, and Dynamical Behaviors. Entropy.

[25] Tokuda H., Hayamizu K., Ishii K., Md A.B.H.S., Watanabe M. (2004). Physicochemical Properties and Structures of Room Temperature Ionic Liquids. 1. Variation of Anionic Species. J. Phys. Chem. B.

[26] Hayamizu K., Tsuzuki S., Seki S., Umebayashi Y. (2012). Multinuclear NMR Studies on Translational and Rotational Motion for Two Ionic Liquids Composed of BF_4_ Anion. J. Phys. Chem. B.

[27] Endo T., Widgeon S., Yu P., Sen S., Nishikawa K. (2012). Cation and Anion Dynamics in Supercooled and Glassy States of the Ionic Liquid 1-Butyl-3-Methylimidazolium Hexafluorophosphate: Results from 13 C, 31 P, and 19 F NMR Spectroscopy. Phys. Rev. B.

[28] Ordikhani Seyedlar A., Stapf S., Mattea C. (2015). Dynamics of the Ionic Liquid 1-Butyl-3-Methylimidazolium Bis(Trifluoromethylsulphonyl)Imide Studied by Nuclear Magnetic Resonance Dispersion and Diffusion. Phys. Chem. Chem. Phys..

[29] Wencka M., Apih T., Korošec R.C., Jenczyk J., Jarek M., Szutkowski K., Jurga S., Dolinšek J. (2017). Molecular Dynamics of 1-Ethyl-3-Methylimidazolium Triflate Ionic Liquid Studied by ^1^ H and ^19^ F Nuclear Magnetic Resonances. Phys. Chem. Chem. Phys..

[30] Bystrov S.S., Matveev V.V., Chernyshev Y.S., Balevičius V., Chizhik V.I. (2019). Molecular Mobility in a Set of Imidazolium-Based Ionic Liquids [Bmim] ^+^ A ^–^ by the NMR-Relaxation Method. J. Phys. Chem. B.

[31] Kruk D., Wojciechowski M., Florek-Wojciechowska M., Singh R.K. (2020). Dynamics of Ionic Liquids in Confinement by Means of NMR Relaxometry—EMIM-FSI in a Silica Matrix as an Example. Materials.

[32] Karagianni M., Gkoura L., Srivastava A., Chatzichristos A., Tsolakis N., Romanos G., Orfanidis S., Panopoulos N., Alhassan S., Homouz D. (2023). Dynamic Molecular Ordering in Multiphasic Nanoconfined Ionic Liquids Detected with Time-Resolved Diffusion NMR. Commun. Mater..

[33] Mossa S. (2018). Re-Entrant Phase Transitions and Dynamics of a Nanoconfined Ionic Liquid. Phys. Rev. X.

[34] Foroutan M., Fatemi S.M., Esmaeilian F. (2017). A Review of the Structure and Dynamics of Nanoconfined Water and Ionic Liquids via Molecular Dynamics Simulation. Eur. Phys. J. E.

[35] Singh M.P., Singh R.K., Chandra S. (2014). Ionic Liquids Confined in Porous Matrices: Physicochemical Properties and Applications. Prog. Mater. Sci..

[36] Buntkowsky G., Breitzke H., Adamczyk A., Roelofs F., Emmler T., Gedat E., Grünberg B., Xu Y., Limbach H.-H., Shenderovich I. (2007). Structural and Dynamical Properties of Guest Molecules Confined in Mesoporous Silica Materials Revealed by NMR. Phys. Chem. Chem. Phys..

[37] Wasyluk L., Peplinska B., Klinowski J., Jurga S. (2002). NMR Studies of the Molecular Dynamics of Tert-Butyl Chloride Confined in the Mesoporous Molecular Sieve MCM-41. Phys. Chem. Chem. Phys..

[38] Le Bideau J., Viau L., Vioux A. (2011). Ionogels, Ionic Liquid Based Hybrid Materials. Chem. Soc. Rev..

[39] Chen S., Kobayashi K., Miyata Y., Imazu N., Saito T., Kitaura R., Shinohara H. (2009). Morphology and Melting Behavior of Ionic Liquids inside Single-Walled Carbon Nanotubes. J. Am. Chem. Soc..

[40] Elverfeldt C.-P., Lee Y.J., Fröba M. (2019). Selective Control of Ion Transport by Nanoconfinement: Ionic Liquid in Mesoporous Resorcinol–Formaldehyde Monolith. ACS Appl. Mater. Interfaces.

[41] Rivera A., Rössler E.A. (2006). Evidence of Secondary Relaxations in the Dielectric Spectra of Ionic Liquids. Phys. Rev. B.

[42] Fujima T., Frusawa H., Ito K. (2002). Merging of α and Slow β Relaxation in Supercooled Liquids. Phys. Rev. E.

[43] Tzialla O., Labropoulos A., Panou A., Sanopoulou M., Kouvelos E., Athanasekou C., Beltsios K., Likodimos V., Falaras P., Romanos G. (2014). Phase Behavior and Permeability of Alkyl-Methyl-Imidazolium Tricyanomethanide Ionic Liquids Supported in Nanoporous Membranes. Sep. Purif. Technol..

[44] Neese F. (2012). The ORCA Program System. WIREs Comput. Mol. Sci..

[45] Neese F. (2018). Software Update: The ORCA Program System, Version 4.0. WIREs Comput. Mol. Sci..

[46] Cowan B. (1997). Nuclear Magnetic Resonance and Relaxation.

[47] Bloembergen N., Purcell E.M., Pound R.V. (1948). Relaxation Effects in Nuclear Magnetic Resonance Absorption. Phys. Rev..

[48] Abragam A. (1989). The Principles of Nuclear Magnetism.

[49] Low F.J., Rorschach H.E. (1960). Nuclear Spin Relaxation in Liquid Helium 3. Phys. Rev..

[50] Kleinberg R.L., Kenyon W.E., Mitra P.P. (1994). Mechanism of NMR Relaxation of Fluids in Rock. J. Magn. Reson. Ser. A.

[51] Shenderovich I.G., Buntkowsky G., Schreiber A., Gedat E., Sharif S., Albrecht J., Golubev N.S., Findenegg G.H., Limbach H.-H. (2003). Pyridine- ^15^  *N* A Mobile NMR Sensor for Surface Acidity and Surface Defects of Mesoporous Silica. J. Phys. Chem. B.

[52] McCall D.W. (1971). Nuclear Magnetic Resonance Studies of Molecular Relaxation Mechanisms in Polymers. Acc. Chem. Res..

[53] Slichter W.P. (1966). NMR Studies of Multiple Relaxations in Polymers. J. Polym. Sci. Part C.

[54] Anderson J.E., Slichter W.P. (1965). Nuclear Spin Relaxation in Solid N-Alkanes. J. Phys. Chem..

[55] van Putte K. (1970). Spin-Lattice Relaxation of CH3 and CH2D Groups in Some Partially Deuterated Alkanes. J. Magn. Reson..

[56] Beckmann P., Ratcliffe C.I., Dunell B.A. (1978). Proton Spin-Lattice Relaxation and Methyl Group Rotation. J. Magn. Reson. (1969).

[57] Popa L.C., Rheingold A.L., Beckmann P.A. (2010). A Proton Spin-Lattice Relaxation Rate Study of Methyl and t-Butyl Group Reorientation in the Solid State. Solid State Nucl. Magn. Reson..

[58] Busch M., Hofmann T., Frick B., Embs J.P., Dyatkin B., Huber P. (2020). Ionic Liquid Dynamics in Nanoporous Carbon: A Pore-Size- and Temperature-Dependent Neutron Spectroscopy Study on Supercapacitor Materials. Phys. Rev. Materials.

[59] Mukhopadhyay R., Alegría A., Colmenero J., Frick B. (1998). Methyl Group Dynamics in Poly(Vinyl Acetate): A Neutron Scattering Study. Macromolecules.

[60] Callaghan P.T. (1993). Principles of Nuclear Magnetic Resonance Microscopy.

[61] McFadden R.M.L., Buck T.J., Chatzichristos A., Chen C.-C., Chow K.H., Cortie D.L., Dehn M.H., Karner V.L., Koumoulis D., Levy C.D.P. (2017). Microscopic Dynamics of Li+ in Rutile TiO_2_ Revealed by 8Li β-Detected Nuclear Magnetic Resonance. Chem. Mater..

[62] Kimmich R., Unrath W., Schnur G., Rommel E. (1991). NMR Measurement of Small Self-Diffusion Coefficients in the Fringe Field of Superconducting Magnets. J. Magn. Reson. (1969).

[63] Day I.J. (2011). On the Inversion of Diffusion NMR Data: Tikhonov Regularization and Optimal Choice of the Regularization Parameter. J. Magn. Reson..

[64] Hassan J., Diamantopoulos G., Gkoura L., Karagianni M., Alhassan S., Kumar S.V., Katsiotis M.S., Karagiannis T., Fardis M., Panopoulos N. (2018). Ultrafast Stratified Diffusion of Water Inside Carbon Nanotubes; Direct Experimental Evidence with 2D *D* – *T* _2_ NMR Spectroscopy. J. Phys. Chem. C.

[65] Angell C.A. (1991). Relaxation in Liquids, Polymers and Plastic Crystals—Strong/Fragile Patterns and Problems. J. Non-Cryst. Solids.

[66] Berthier L., Biroli G. (2011). Theoretical Perspective on the Glass Transition and Amorphous Materials. Rev. Mod. Phys..

[67] Garca-Coln L.S., del Castillo L.F., Goldstein P. (1989). Theoretical Basis for the Vogel-Fulcher-Tammann Equation. Phys. Rev. B.

[68] Ordikhani Seyedlar A., Stapf S., Mattea C. (2019). Nuclear Magnetic Relaxation and Diffusion Study of the Ionic Liquids 1-Ethyl- and 1-Butyl-3-Methylimidazolium Bis(Trifluoromethylsulfonyl)Imide Confined in Porous Glass. Magn. Reson. Chem..

[69] Kruk M., Jaroniec M., Ko C.H., Ryoo R. (2000). Characterization of the Porous Structure of SBA-15. Chem. Mater..

[70] Vila J., Fernández-Castro B., Rilo E., Carrete J., Domínguez-Pérez M., Rodríguez J.R., García M., Varela L.M., Cabeza O. (2012). Liquid–Solid–Liquid Phase Transition Hysteresis Loops in the Ionic Conductivity of Ten Imidazolium-Based Ionic Liquids. Fluid Phase Equilibria.

[71] Imanari M., Uchida K., Miyano K., Seki H., Nishikawa K. (2010). NMR Study on Relationships between Reorientational Dynamics and Phase Behaviour of Room-Temperature Ionic Liquids: 1-Alkyl-3-Methylimidazolium Cations. Phys. Chem. Chem. Phys..

[72] Imanari M., Nakakoshi M., Seki H., Nishikawa K. (2008). 1H NMR Study on Reorientational Dynamics of an Ionic Liquid, 1-Butyl-3-Methylimidazolium Bromide, Accompanied with Phase Transitions. Chem. Phys. Lett..

